# Host Transcription Factors in Hepatitis B Virus RNA Synthesis

**DOI:** 10.3390/v12020160

**Published:** 2020-01-30

**Authors:** Kristi L. Turton, Vanessa Meier-Stephenson, Maulik D. Badmalia, Carla S. Coffin, Trushar R. Patel

**Affiliations:** 1 Alberta RNA Research and Training Institute, Department of Chemistry and Biochemistry, University of Lethbridge, 4401 University Dr. W., Lethbridge, AB T1K 3M4, Canada; kristi.turton@uleth.ca (K.L.T.); vmeierstephenson@gmail.com (V.M.-S.); maulik.badmalia@uleth.ca (M.D.B.); 2 Department of Microbiology, Immunology and Infectious Diseases, Cumming, School of Medicine, University of Calgary, 2500 University Dr. N.W., Calgary, AB T2N 1N4, Canada; cscoffin@ucalgary.ca; 3 Department of Medicine, Division of Gastroenterology and Hepatology, University of Calgary, 1403 29 St. N.W., Calgary, AB T2N 2T9, Canada; 4 Discovery Lab, Faculty of Medicine & Dentistry, and the Li Ka Shing Institute of Virology, University of Alberta, 6-010 Katz Center for Health Research, Edmonton, AB T6G 2E1, Canada

**Keywords:** hepatitis B virus (HBV), viral replication, transcription factors, covalently closed circular DNA (cccDNA), host–viral interactions

## Abstract

The hepatitis B virus (HBV) chronically infects over 250 million people worldwide and is one of the leading causes of liver cancer and hepatocellular carcinoma. HBV persistence is due in part to the highly stable HBV minichromosome or HBV covalently closed circular DNA (cccDNA) that resides in the nucleus. As HBV replication requires the help of host transcription factors to replicate, focusing on host protein–HBV genome interactions may reveal insights into new drug targets against cccDNA. The structural details on such complexes, however, remain poorly defined. In this review, the current literature regarding host transcription factors’ interactions with HBV cccDNA is discussed.

## 1. Introduction

The hepatitis B virus (HBV) is estimated to chronically infect 257 million people worldwide [1]. Chronic HBV infection can promote liver fibrosis and cirrhosis and is also independently associated with hepatocellular carcinoma [2,3,4,5,6,7]. HBV persists in part due to its resilient minigenome, the covalently closed circular DNA (cccDNA), which resides in the nucleus of the infected cells and acts as the template for all HBV RNA production. The HBV genome can integrate and exist within the host genome [7,8]. Current therapies against HBV, including the nucleoside/tide analogues (NAs), are effective at targeting viral reverse transcriptase activity and reducing viral replication but they do not directly target the cccDNA [9,10], often leading to relapse of viremia once therapies are stopped. Eradication of HBV cccDNA is essential to ultimately achieve a virological or sterilizing cure of HBV infection.

HBV belongs to the *Hepadnaviridae* family and has one of the smallest, compact viral genomes (~3.2 kb), that encodes five RNA transcripts and seven proteins. Its small size limits the number of viral proteins or nucleic acid sequences available for targeting to inhibit HBV replication. A feature common to all viruses, including HBV, is the requirement of their genome to interact with host proteins to complete the viral replication cycle. Targeting this interaction between the HBV cccDNA and host proteins could represent a potential therapeutic target.

**Figure 1:**
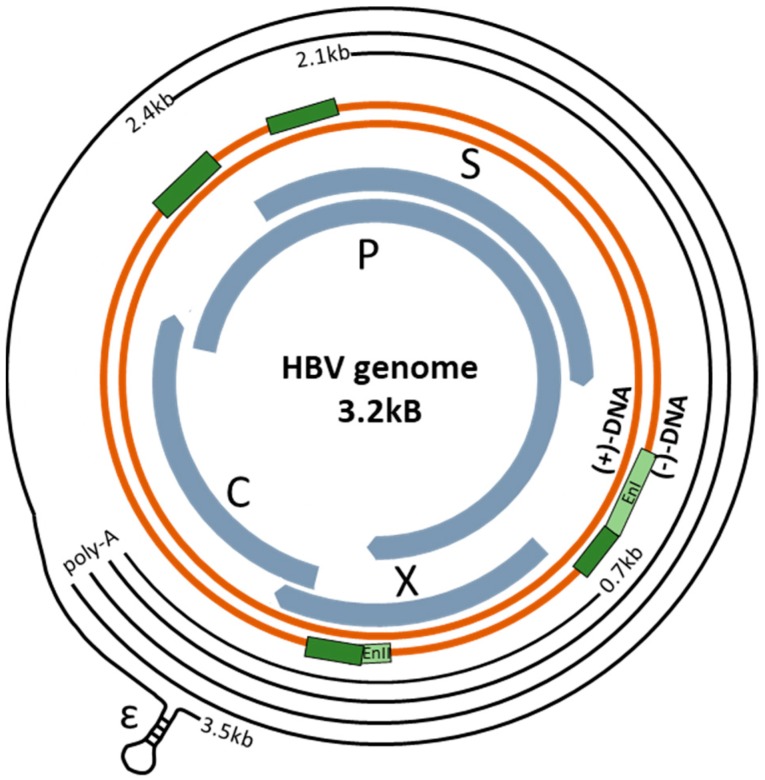
Schematic of the hepatitis B virus (HBV) circular genome (dark orange parallel lines). The outer black lines represent the RNA transcripts produced, all ending in the poly-A tail. The promoter regions are denoted by the dark green rectangles preceding each of these transcripts, and the Enhancer regions I and II, (EnI and EnII) are denoted by the light green rectangles. The thicker inner arrows represent the open reading frames, encoding for the C (core), P (polymerase), S (surface), and X proteins. The encapsidation signal, ԑ, is also noted.

Transcription factors function by interacting with DNA sequences upstream of the gene to be transcribed, i.e., promoter regions, to control transcriptional activity, where regulation can take the form of initiation and/or inhibition of transcription [11,12]. The messenger RNA transcripts of the HBV genome (including its pre-genomic RNA [pgRNA] replication intermediate) are under the control of four promoter regions—preS1, preS2, X-promoter, and pre-core/core promoters—and two enhancers, ENI and ENII (Figure 1). Several host transcription factors are recruited to these sites in cccDNA to enable HBV replication [3,11,13]. These transcription factors can be classified as those unique to hepatocytes or present in many types of cells, i.e., ubiquitous transcription factors. Understanding the interactions between host transcription factors and the HBV genome will enhance our knowledge of HBV replication. 

In this review, we summarize the literature on the various host proteins known to act as transcription factors for HBV replication. We highlight their functions in the host cell, delineate whether selective inhibition is feasible and include structural or sequence-specific information relevant to the interaction with HBV.

## 2. The HBV Genome and Control of Viral Replication

HBV belongs to the *Hepadnaviridae* family and, as a virion, contains partially double-stranded relaxed circular DNA (rcDNA) (Figure 1). Its virion has one of the smallest genomes at ~3.2 kbp and is contained within the nucleocapsid core that is in turn surrounded by a lipoprotein envelope composed of surface glycoproteins. The primary target of HBV is liver hepatocytes where HBV pre-S1 protein interacts with the sodium taurocholate cotransporting polypeptide (NTCP) and the epidermal growth factor receptor to enter into hepatocytes [14,15]. Once in the host cells, the viral envelope disintegrates and the nucleocapsid localizes into the nucleus with the help of HBV core protein (contains nuclear localization signal) [16]. In the nucleus, the HBV rcDNA undergoes processing to remove the polymerase from the minus strand, remove an RNA oligomer in the positive strand, and synthesize the RNA fragment to a complete positive strand to ultimately form covalently closed circular DNA (cccDNA) [17]. The cccDNA is then bound to histones creating the compact, stable form of the virus from which all the viral RNA transcripts are produced, including pgRNA [18,19]. The pgRNA (positive strand) is transcribed from cccDNA and exported from the nucleus to the cytoplasm where viral proteins are synthesized using host cell machinery. It is estimated that median 1.5 copies of cccDNA can be found in each individual hepatocyte [20]. However, the viral and host factors that regulate the formation of cccDNA and control cccDNA copy numbers remain elusive. 

The HBV genome is comprised of four overlapping open reading frames (ORFs) that code for the surface (S), core (C), polymerase (P) and X proteins (Figure 1) [4,5,21]. The HBV S proteins, comprising of large (L), middle (M), and small (S) proteins make up the envelope of the virion; the preC-C protein that encodes the viral nucleocapsid; and the viral P or reverse transcriptase, which aids genome synthesis [3,5,22,23,24]. Additionally, the pre-C protein can undergo C-terminal truncation forming the accessory protein HBV e antigen (HBeAg) [25]. The X protein, also known as HBx, aids viral replication by interacting with and transactivating host factors to prevent an antiviral response or by facilitating proviral cellular activity [26,27,28]. For example, Decorsière et al. determined that HBx interacts with host proteins in the ubiquitin-proteasome system in order to degrade viral suppressor proteins [29,30]. Additionally, HBx binds to the cccDNA which has been hypothesized to modulate HBV transcription possibly by modifying the epigenetic modifications that occur in vivo [31]. Further, it has been found to play important roles in the epigenetic control of cccDNA processing and stability via the recruitment of histone-modifying enzymes [31]. There are also cis-elements present within the HBV genome known as enhancer elements (EN) and negative regulating elements (NRE), which regulate overall viral promoter activity [5,17,32]. The two enhancer regions (ENI and ENII) are of interest due to the high amount of transcription binding activity in this region [32]. 

This review will summarize the research on HBV transcription factors, broadly classified into ubiquitous and liver-enriched transcription factors. The current understanding of interactions between the cccDNA genome and host transcription factors are discussed in the context of future antiviral strategies to achieve an HBV cure.

## 3. Transcription Factors

**Figure 2:**
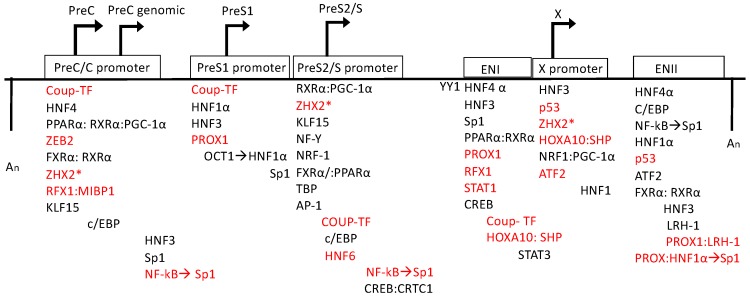
Relative positions of the binding sites of host transcription factors on the HBV genome. Promoter and enhancer sites are represented by the boxes and the directional arrow indicates the transcript produced. The arrows from one transcription factor to another represents an event in which one factor influences the binding activity of another. The two An sites represent the polyadenylation sites in the linearized genome schematic. Transcription factor activity has been indicated in which red represents inhibition of HBV transcription and black indicates activation of transcription. The lists of transcription factors are positioned to represent where they bind to the genome where they are generally positioned to 5′ or 3′ of the promoter/enhancer region (not to scale). Asterisks indicate that the specific positioning of the transcription factor is not yet known.

Host transcription factors discussed in the current review can be classified as either ubiquitous (generally found in all cell types) and liver-enriched (unique to hepatocytes). As the HBV cccDNA resides in the nucleus of hepatocytes, both categories are candidates for aiding viral transcription, and therefore potential therapeutic targets (Figure 2). 

### 3.1. Ubiquitous Transcription Factors

Ubiquitous transcription factors are found in essentially all cell types and are associated with various cellular processes such as cell proliferation, cell differentiation and host immune response. They can give an insight on viral replication in all host cell types, and the clinical/disease consequences of persistent viral infection. The transcription factors discussed in this review are summarized in Table 1.

#### 3.1.1. Nuclear Transcription Factor Y

Nuclear transcription factor NF-Y is composed of three protein subunits, NF-YA, NF-YB and NF-YC. With the formation of the heterotrimer via histone fold domains in each subunit, NF-Y binds to the CCAAT binding motif of genomic DNA [83,84]. It is associated with multiple cellular processes such as cell proliferation, the cell cycle, and responds to cellular events such as DNA damage [85]. NF-Y is also linked with the posttranslational modifications of histone proteins by recruiting enzymes that both methylate and acetylate histone proteins [84,86]. In HBV infection, NF-Y has been confirmed to bind to the S promoter region in the CCAAT binding domain and initiate HBV S protein transcription in both in vitro and in vivo models [4]. A mutant version lacking the binding domain displayed a 10-fold decrease in HBV surface antigen (HBsAg) expression compared to wildtype infection, suggesting that NF-Y is essential for HBV replication [4]. NF-Y also aids shuttling chaperone proteins to the endoplasmic reticulum, possibly triggered by the accumulation of HBV S proteins in an unfolded state, providing additional evidence for the role of this factor in HBV replication [87].

#### 3.1.2. Nuclear Factor kappa B

Nuclear Factor kappa B (NF-κB) is a sequence-specific DNA binding protein (GGGRNYYYYC), where R represents a purine, Y represents a pyrimidine and N represents any nucleotide) that can bind to DNA as a homodimer or a heterodimer [88]. NF-κB is consisting of two subunits p50 and p65, which dimerize via the binding of the Rel homology regions in each subunit and interacts with palindromic sites in the DNA, known as kB sites [89,90]. 

NF-κB acts as a regulating element for both innate and adaptive cellular immune responses. It is associated with cellular processes such as angiogenesis, apoptosis, cell proliferation, and migration [13,88]. In the host cell, NF-κB expression is induced by infection with microbial pathogens, oxidative stress or DNA damage [88]. Once activated, it binds to promoters to initiate transcription of immune-associated proteins such as interleukin 6 (IL-6) [91]. Previous studies in Huh7 and HepG2 cell lines have revealed that NF-κB is able to inhibit transcription of the HBV genome [13,92] due to its activation after the induction of oxidative stress by HBx [42]. This activation may be a residual effect as HBx is proposed to modulate host cell transcription through interactions with Ras and PKC pathways [93]. Although the response from NF-κB is to prevent HBV infection, constant activation of interleukin proteins and cytokine networks through the response of NF-κB can cause tissue damage and oncogenesis [93,94].

#### 3.1.3. Specificity Protein 1

**Figure 3:**
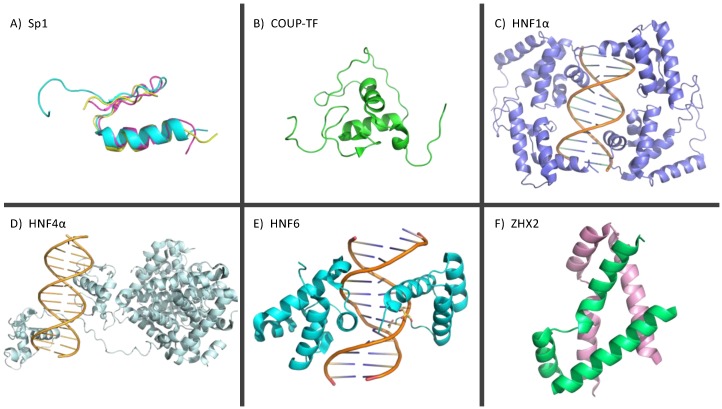
An overview of high-resolution structures of transcription factors. (**A**) An overlay of high-resolution structures of three zinc finger domains of Sp1 [53]. (**B**) A structure of COUP-TF zinc finger domain [pdb: 2EBL]. (**C**,**D**) High-resolution structures of HNF1α [201] and HNF4α [202] bound to the major groove of DNA. In both cases, cccDNA DNA sequences (5′-GTTAATNATTAAC-3′ recognized by HNF1α and 5′-CAGAGGNCAAAGTCCA-3′ by HNF4α) were used to calculate 3 dimensional structures employing 3D-DART web server [203]. (**E**) High-resolution structure of mouse HNF-6α DNA-binding domain bound, which is identical to human HNF-6α DNA-binding, with transthyretin (TTR) promoter [204]. (**F**) Dimeric structure of homeodomain 2 of ZHX2 protein [205].

One of the most studied transcription factors in HBV studies is Specificity protein 1 (Sp1), a Krüppel-like factor (a family of transcription factors with zinc finger domains) that plays critical roles in gene expression in early stages of development [95]. Sp1 is also associated with a range of cellular processes such as angiogenesis, apoptosis and the cell cycle [96]. Sp1 is comprised of three zinc finger domains (Figure 3A), which are located downstream of one DNA-binding domain within its protein structure, these domains are flanked by auxiliary domains responsible for oligomerization and inhibition. It should be noted that these functions are putative and detailed structural-functional studies will shed more light on Sp1′s role in HBV replication [23,52,53]. Figure 3A represents the solution structures of three zinc finger domains determined using the nuclear magnetic resonance technique [52]. Sp1 has several binding motifs in the human genome, including GC-rich regions and CACCC motifs and can help regulate the expression of both TATA-containing and TATA-less genes [97,98,99].

In HBV, Sp1 has been demonstrated to bind to several sites in the HBV genome including the core promoter, the PreS1/S2 promoter and the ENII region [13,23] and has been reported to regulate HBV gene expression during viral infection. In this study, the Sp1 binding regions in HBV were mapped by introducing single mutations in the putative binding regions (G-to-A at nucleotide 1748 and C-to-A at nucleotide 1630 respectively) and the plasmids containing the mutants were transfected into Huh7 hepatoma cells [23]. Both introduced mutations had caused a decrease in transcription of C and the latter mutation caused a simultaneous increase in S and X transcription [23]. This study shows that Sp1 interaction with cccDNA has a direct impact on HBV replication. 

#### 3.1.4. Chicken Ovalbumin Upstream Promoter Transcription Factor

Chicken Ovalbumin Upstream Promoter Transcription Factor (COUP-TF) is also known to be an orphan nuclear receptor and is part of the steroid/thyroid family [100]. The COUP-TF contains a ligand-binding domain, two activation domains that allow for the binding of essential co-factors and a DNA-binding domain that contains two zinc finger domains (Figure 3B) [39,101]. The two C2H2 zinc fingers are found to be close to each other spatially. Typically, the COUP-TF dimerizes to recognize and bind the GGTCA half repeat motif. This motif has two of the same repeats separated by a variable six nucleotide gap [100,102]. The two-finger zinc finger domains work together to recognize and bind to the DNA, which is a characteristic of other steroid/thyroid hormone receptors [100]. DNA binding can only occur when the COUP-TF proteins are not bound to the receptor-ligand [100]. 

COUP-TF is primarily associated with the hormone response by both binding to secreted hormones and interacting with other hormone receptors such as the Retinoid X receptor and the thyroid hormone receptor [13,103,104]. It has additional roles in organ development, neural development, and cell migration/differentiation [105,106]. In terms of HBV replication, COUP-TF is known to repress transcription in HepG2 cells by interacting with the ENI element and represses C and S transcription levels [107]. There are also studies suggesting that COUP-TF is potentially able to repress ENII activity [108]. The binding to the ENI site is dependent on the levels/ratio of hepatocyte nuclear factor 4 present (HNF4α), as these two transcription factors compete for binding to the promoter regions described [109].

#### 3.1.5. CCAAT Enhancer Binding Protein Family

CCAAT is a binding motif found in promoter regions of various eukaryotic proteins. A family of proteins that bind this motif is called the CCAAT Enhancer Binding Protein (C/EBP) family and are characterized as having leucine zipper binding domains [110]. C/EBPs proteins (six members—C/EBPα–C/EBPζ) either interact as heterodimers or homodimers to bind the CCAAT motifs in DNA [35]. C/EBP transcription factors are associated with functions in the liver that include metabolism, hormone control, and hepatocyte regeneration [111] as well as influence functions of myeloid-derived suppressor cells [112]. 

C/EBP can bind to the ENII site in the Pre-core promoter of the HBV genome in HepG2 cells which are facilitated by interactions with the HBx protein [113,114]. The binding of C/EBP can positively or negatively regulate transcription based on expression levels where low expression induces transcription, and higher levels are repressive [114,115]. In Huh7, HepG2 and HeLa cell lines, The C/EBP can also bind to the S promoter to enhance transcription [116]. Researchers have studied how RNA may be able to affect virulence through interactions with C/EBP [117,118]. For example, Sarkar et al. [118] illustrated that microRNA-155, an RNA associated with the innate immune response, is capable of inhibiting C/EBP-β and therefore causing a decrease in viral load.

#### 3.1.6. Transcription Factor IIB

Transcription Factor IIB (TFIIB) is a part of the transcription initiation complex in the host cells. It ensures proper orientation of the DNA-RNA duplex to facilitate RNA synthesis [119]. The active site of TFIIB contains zinc finger binding domains (with the amino acid structure of CXHXCXC) which help bind to promoter regions of DNA [59].

The HBx protein interacts with TFIIB protein facilitating viral replication, where HBx acts by first activating TFIIB, causing the transcription initiation complex to bind to the promoter regions [60,120]. Haviv et al. confirmed the direct binding of HBx with TFIIB using coimmunoprecipitation techniques [120] by demonstrating that compared to wild type, TFIIB zinc finger mutants were found to not coimmunoprecipitate with HBx confirming disruption of binding. This interaction is critical for the coactivation ability of HBx [121]. TFIIB can associate with HBx as well as with another RNA polymerase (RPB5), which upregulates viral RNA transcription levels and higher viral replication [60,122].

#### 3.1.7. Zinc-Finger E-Box Binding Homeobox 2

Zinc-finger E-box binding homeobox 2 (ZEB2) is a ubiquitous transcription factor that plays critical roles in TGF-β, and Bone Morphogenetic Protein (BMP) signaling pathways [123,124]. It is also crucial in embryonic development [125] and the development of the neural crest [126,127]. ZEB2 has two zinc finger clusters that bind to CACCT sequences and inhibit transcription [128,129]. Each of the two zinc finger clusters has three zinc finger domains, most having the C2H2 zinc finger motifs [128]. ZEB2 was identified as an HBV core promoter interacting protein in HepG2.2.15 and HepAD38 cell lines using Chromatin immunoprecipitation (ChIP) assays and was demonstrated to inhibit HBV replication [65].

#### 3.1.8. Tumor Protein 53

Tumor protein 53 (also known as P53) is a crucial tumor suppressor protein that is involved with cell proliferation, cell death, DNA damage, oxidative stress, hypoxia, etc. and acts as a transcription factor for several genes including CDKN1A and BAX [130,131]. p53 binds DNA as homotetramer which binds to one DNA half-site called a response element (RRRCWWGYYY), therefore indicating that a full response element is bound by two homotetramers [130,132]. The homotetramer structure is facilitated by the tetramerization domain on the C-terminus of the protein [130]. The DNA-binding domain also contains two clusters of zinc fingers which each contain two zinc finger motifs [133].

p53 inhibits HBV replication by interacting with the ENII and the X promoter region [61,62,63]. However, HBx can alter the inhibitory effects of p53 by directly or indirectly interacting with p53 [134,135]. ChIP-sequencing experiments using HepG2 cells suggested that p53 had altered binding activity in the presence of HBx. Recruitment of host DNA is decreased by the disruption of P53-TBP/TFAP2A-RB1 protein complexes by HBx but the recruitment of host DNA is increased with p53 associated transcription factors such as Sp1 and c-JUN, which alters site binding in favor for HBV replication [135].

#### 3.1.9. Regulatory Factor Box 1

Regulatory Factor Box 1 (RFX1) is a ubiquitous transcription factor that regulates several genes such as MHC class II proteins, interleukin-5 receptor α5, and c-Myc indicating its importance in the host immune response and cell proliferation [136]. RFX1 binds to DNA using a winged-helix (helix-turn-helix) sequence and binds DNA in a sequence-specific manner (GTNRCC(0-3N)RGYAAC) [137].

RFX1 is able to inhibit HBV replication by binding to the ENI site and core promoter [48,49,50]. Julien et al. employed antisense oligonucleotides to inhibit RFX1, allowing for HBV surface antigen expression in HepG2 cell lines [138]. Interestingly, ENI binding does not occur in non-hepatic cell lines, suggesting that RFX1 is interacting with liver-enriched transcription factors to inhibit HBV replication at this site [138]. In order to bind the core promoter of the HBV genome, RFX1 binds to a factor called the c-Myc Intron Binding Protein 1 (MIBP1) [49].

#### 3.1.10. Homeobox A10

Homeobox A10 (HOXA10) is a transcription factor critical in processes such as endometriosis, embryogenesis, and cell differentiation [139,140,141]. HOXA10 is able to bind to DNA (AA(AT)TTTTATTAC) through its homeobox domain; a helix-turn-helix motif that is abundantly found in prokaryotic DNA-binding proteins [142]. 

HOXA10 can inhibit HBV replication through binding of the X and ENI regions of the genome and has been confirmed in HepG2, HepG2.2.15, HepG2-NTCP, primary hepatocytes and mice [40]. Interestingly, binding of HOXA10 to ENI competes for binding with Signal Transducer and Activator of Transcription 3 (STAT3), disrupting STAT3 initiated transcription [40]. Disruption of initiation via STAT3 also occurs indirectly as HOXA10 interacts with the MAPK/STAT3 pathway. The recruitment of small heterodimer partner (SHP) preventing STAT3 phosphorylation that allows for the functional conformation [40].

#### 3.1.11. Octamer Binding Protein 1

Octamer binding protein 1 (Oct1) is a ubiquitous transcription factor that recognizes a DNA sequence called the octamer motif (ATGC(A/TA)AAT). It can also recognize similar sequences with the addition of a second Oct1 protein [143,144]. Oct1 has a DNA-binding domain called the POU domain that contains a POU-specific domain and a POU-homeodomain [145]. Oct1 is critical for a variety of biological processes such as T-cell differentiation, tissue development, and oxidative and metabolic stress responses [146,147,148]. 

Oct1 can bind to the PreS1 promoter in the HBV genome in HepG2 cells and HeLa cells and works with HNF1 as an essential co-activator to activate transcription [46]. Additionally, the HBx protein interacts with the Oct-1 protein that is in complex with other preinitiation factors in order to bind the U6 snRNA promoter and initiate transcription [149].

#### 3.1.12. Nuclear Respiratory Factor 1

Nuclear respiratory factor 1 (NRF1) is a ubiquitous transcription factor related to mitochondrial biogenesis and function and plays a role in apoptosis through the mitochondrial associated genes [150,151,152]. NRF1 interacts with the consensus DNA sequence (YGCGCAYGCGCR) DNA via its two zinc finger domains [153,154]. NRF1 has been shown to recruit Peroxisome proliferator-activated receptor-gamma coactivator (PGC-1α) but this is not essential for binding to the DNA [45]. 

Although the endogenous level of NRF1 is not limiting to HBV replication, it has been demonstrated to bind to the X promoter and activated gene transcription as determined in HepG2 cell lines [44].

#### 3.1.13. Signal Transducer and Activator of Transcription 1

Signal Transducer and Activator of Transcription 1 (STAT1) is a ubiquitous protein, which is highly expressed in lymphocytes. It is involved with processes such as infection defense, immune tolerance, cell proliferation and apoptosis [155,156]. STAT proteins bind as dimers or higher-order structures in order to bind specific DNA sequences (NATTTCCNGGAAAT) [157,158]. 

Stat1 binds to the ENI/X promoter region of the genome [55]. However, HBx actively prevents STAT1 activity by disrupting its ability to activate interferons. Type I interferon induction is blocked upstream in the signaling pathway as seen in Hep2.2.15 cell lines [159]. HBx is able to prevent interferon transcription by inhibiting the nuclear translocation of STAT1 [54]. It should be noted that HBx can disrupt numerous upstream factors such as kinase epsilon (IKKε) and interferon regulatory factor 3 (IRF3) [54].

#### 3.1.14. Signal Transducer and Activator of Transcription 3

Signal Transducer and Activator of Transcription 3 (STAT3) is also a ubiquitous transcription factor that is a part of the JAK/STAT pathway. STAT3 influences the host immune response, cell cycle regulation, growth factors regulation, cell transformation, and many other cellular processes [160,161,162]. In order to bind DNA, Tyr-709 of STAT3 is phosphorylated followed by the formation of a homodimer via its SH2 domain before entering the nucleus and binding to the consensus sequence TTCCCGGAA [163]. 

STAT3 can also promote HBV replication by interacting with HNF3 that is bound to the ENI site of the HBV genome [25,56]. The activation of STAT3 and an increase in STAT3 activator IL-6 was seen in studies with HepG2 and HepG2.2.15 cells. The silencing of STAT3 via shRNA leads to inhibition of HBV replication in both cell lines and mice [56].

#### 3.1.15. Activator Protein 1

Activator protein 1 (AP-1), a ubiquitous transcription factor, is associated with cellular processes such as cell proliferation, apoptosis, cell differentiation and angiogenesis [164,165,166]. AP-1 refers to dimeric complexes that include transcription factor families such as JUN, FOS, ATF, and MAF. Both the DNA binding and dimerization of these AP-1 complexes are facilitated by bZIP leucine-zipper domains [164]. Once dimerized the complex can then recognize the DNA consensus sequence TGA(G/C)TCA [167]. 

AP-1 is activated by the JNK pathway [168] and can also be activated indirectly by HBx which binds to Jak1 or JAB1 in vivo [134,169]. Once activated, AP-1 is able to activate the PreS2/S region of the HBV genome as determined in HepG2 and HeLa cells [34,170].

#### 3.1.16. Prospero-Related Homeobox Protein 1

Proper-related homeobox protein (PROX1) is also a ubiquitous transcription factor that influences embryonic development, cell fate, neural and tissue development, and regulates the circadian rhythm [171,172,173]. DNA binding is facilitated through its homeodomain which has a helix-loop-helix-turn-helix structure which can bind to the consensus sequences (A/C) AAGN(C/G/T) or CA(C/G/T)NN(C/G)Y [171,174].

PROX1 interacts with the DNA binding and ligand-binding domain of the liver receptor homolog-1 (LRH-1) [175]. When in complex with LRH-1, ENII downregulation occurs. Overall, Prox1 has been identified to bind to the ENI, ENII and PreS1 promoter and repress HBV replication. Interestingly, PROX1 can also interact with HNF1α and disrupt Sp1 activity when studied in HepG2, Huh7 and HeLa cells [174].

#### 3.1.17. TATA Box Protein

TATA box protein (TBP) is a ubiquitous transcription factor that is crucial for host transcription initiation and is functional once bound to the transcription factor IID (TFIID) complex [176]. The TBP has a saddle-like structure in its DNA-binding domain that allows binding to the TATA consensus sequence (TATAa/tAa/t) [177,178].

The TBP protein interacts with TATA-like sequences in the PreS2/S promoter region in the HBV genome and enhances HBV replication as studied in HepG2 and Huh7 cell lines [58]. The HBx protein has also been shown to interact with TBP on an ATP-dependent basis in vitro to further facilitate HBV replication [179].

#### 3.1.18. Yin Yang 1

Yin Yang 1 (YY1) is a ubiquitous transcription factor that has roles in development, cell differentiation, proliferation and apoptosis [180,181]. YY1 binds DNA through its Zinc finger motifs and recognizes the DNA sequence CGCCATNTT [180,182]. 

YY1 has been shown to bind to cccDNA in HepG2 cell lines, binding to an integration sequence next to the direct repeat region 1 (DR1) in the HBV genome [55,64,183]. However, this binding does not affect HBV transcription but instead has a role in the integration of the HBV genome into the host genome [184]. Transcriptional activity is not directly with the HBV genome. Studies have indicated that YY1 regulates other signaling pathways that facilitate HBV replication. Shang et al. discovered that YY1 activates the sex-determining region Y box 4 protein in HepG2 cells with opposing results in control cells, suggesting a role in HBV replication [185].

#### 3.1.19. Activating Transcription Factor 2

Activating Transcription Factor 2 (ATF2) is critical for biological processes including cell proliferation, inflammation, apoptosis, neurological development and skeletal remodeling, acetyltransferase activity, and DNA damage [186,187,188]. ATF2 binds to the cAMP response element (cre) with the consensus sequence TGACGTCA with its C-terminal DNA-binding domain which contains a bZip leucine zipper motif [189]. It can also bind to the AP-1 consensus sequence [190].

ATF2 competitively binds with AP-1 to the ENI promoter region and is able to repress the transcription of X gene in HepG2 cells. Choi et al. determined that with transfection of a vector containing AFT2 into HepG2 cells caused a decrease in JUN/FOS binding to the ENI site [33].

#### 3.1.20. cAMP Response Element-Binding Transcription Factor

cAMP response element-binding transcription factor (CREB) is a ubiquitous transcription factor that is associated with cellular processes gluconeogenesis, lipid metabolism, cell proliferation and neuronal plasticity [191,192,193]. CREB binds to the cre element within the genome via its bZip motif [194]. 

Faktor et al. demonstrated that CREB interacts with the ENI region of HBV [37]. Subsequent studies by Kim et al. suggested that the cre sequence in HBV is highly conserved and interaction of HBV genome with CREB is essential for HBV replication [36]. CREB can also facilitate the binding of the CREB-regulated transcriptional coactivator 1 protein (CRTC1) to the PreS2/S site and positively influence HBV transcription. Contrarily, inactive CRT1 protein at high levels was shown to inhibit HBV transcription in HepG2 cells [195].

#### 3.1.21. Small Heterodimer Partner

Small heterodimer partner (SHP) is an orphan nuclear receptor associated with the regulation of bile acid synthesis, lipid metabolism, cancer development, microRNA expression and innate immune system [196,197,198,199]. Interestingly, SHP does not contain a DNA-binding domain, suggesting that it acts as a cofactor, facilitating binding with HBV components [51].

HNF4α bound to SHP was demonstrated to repress HBV replication in HepG2 cells as well as non-hepatic cell lines [51]. Reese et al. concluded that SHP and its corepressor FXRα have a limited role in HBV replication [200]. Additionally, SHP is also recruited by HOXA10, leading to suppression of p38 MAPK/STAT3 pathway and HBV replication [40].

### 3.2. Liver-Enriched Transcription Factors

HBV is a hepatotropic virus that gains entry into hepatocytes via the host’s bile salt receptor- sodium taurocholate co-transporting polypeptide (NTCP) in complex with the epidermal growth factor, when interacting with the pre-S1 domain of the HBV large envelope protein [14,206,207,208,209]. As such, liver-specific transcription factors have been implicated in supporting HBV’s replication cycle. 

#### 3.2.1. Hepatocyte Nuclear Factor 1α

Hepatocyte nuclear factor 1 alpha (HNF1α) is a part of the HNF1 sub-family [210]. HNF1α has an N-terminal POU domain, a DNA binding homeodomain and an N-terminal dimerization domain [211]. The HNF1α protein interacts with other HNF1 family proteins to form homodimers or heterodimers [212,213]. The homodimer/heterodimer structure allows for binding to the DNA which is facilitated by the POU domain (Figure 3C) [210,211]. HNF1α regulates tissue development and associated processes, maintains amino acid levels, and gluconeogenesis [214,215,216].

The POU homeodomain in HNF1α is suggested to bind to the PreS1 promoter region of the HBV genome [46]. Additionally, HNF1α interacts with HBx, the core promoter and the ENII element [71,72,217]. HNF1α’s interaction with the ENII element is crucial for an efficient HBV replication in hepatic cell lines; activation of the ENII element is prevented if the HNF1α is knocked down [218]. However, in vivo studies suggest that the absence of HNF1α does not prevent HBV replication [218]. Furthermore, high HNF1α levels enhance expression of the NF-κB transcription factor which in turn inhibits HBV genome transcription [219]. Additionally, it was demonstrated that its overexpression leads to inhibition of HBV gene expression, whereas its absence enhances expression [219]. 

#### 3.2.2. Hepatocyte Nuclear Factor 3

Hepatocyte nuclear factor 3 (HNF3 also known as FOXA) encompasses a subfamily of transcription factors that includes HNF3α, β and γ [220]. HNF3 transcription factors contain a conserved winged-helix DNA-binding region. The third helical structure in this domain recognizes the genomic DNA and binds within the major groove of the DNA helix [220]. The HNF3 proteins primarily regulate metabolic cellular processes such as carbohydrate and lipid metabolism, and fatty acid oxidation [221,222,223].

The HNF3α, β and γ proteins were shown to bind with ENI and ENII and increase their activity in Huh7, HepG2 and HeLa cell lines [38,73]. Additionally, HNF3 interacts with the PreS1 promoter region in HepG2 cells [74]. However, contradicting evidence has emerged in terms of how HNF3 can regulate the activity. HBV replication studies in HepG2 cells indicated enhanced viral gene expression while expression in SK-Hep1 cells suggested inhibited HBV replication [224,225], highlighting the need for additional mechanistic studies.

It is hypothesized that HNF3 can unwind HBV cccDNA and facilitate viral transcription by allowing the binding of other transcription factors [226]. Future studies on the HNF3 function will provide a more in-depth understanding of HNF3′s role in HBV replication. 

#### 3.2.3. Hepatocyte Nuclear Factor 4α

Hepatocyte nuclear factor 4α (HNF4α) is a nuclear receptor that regulates HBV transcription [227]. Similar to HNF1α, HNF4α contains a POU domain consisting of zinc finger regions that allow for DNA binding (Figure 3D) [228]. HNF4α is associated with lipid and carbohydrate metabolism, inflammation as well as embryogenesis [229,230,231,232]. Although found in other cell types, HNF4α is expressed in hepatocytes and is upregulated upon HBV infection [233]. HNF4α is a positive regulator of HBV infection that is recognized to interact with the ENI, ENII (in Huh7 cells) and preS1 regions (HepG2 cells) [72,75,76,77]. Researchers have explored the potential of reducing infection by lowering HNF4α levels in mice, which revealed that siRNA knockdown of HNF4α prevents HBV replication and reduced pre-genomic RNA levels, but also impaired glucose homeostasis and growth of the cells [218,234]. Wang et al. demonstrated that estrogen receptor alpha (ERα), when bound to HNF4α, leads to lower expression levels of HBV genes [235]. The binding of ERα was confirmed to affect the binding affinity of HNF4α for the ENI site and is a proposed mechanism for viral load and infection incidence disparity between males and females [235].

#### 3.2.4. Hepatocyte Nuclear Factor 6

Hepatocyte nuclear factor 6 (HNF6) is present in various cell types such as pancreatic cells and the gallbladder, but the highest levels can be found in hepatocytes [236]. HNF6 contains two DNA-binding domains known as the homeodomain and single cut domain, where the homeodomain primarily binds the DNA and the single cut domain binds co-activating proteins for initiation of transcription [237]. Figure 3E represents a high-resolution structure of mouse HNF6 bound with transthyretin promoter [204]. It plays key roles in many critical cellular processes such as pancreas development, suppression of cancer cell growth, cell differentiation, morphogenesis, lipid metabolism, and growth hormone production [238,239,240,241,242]. 

Hao et al. demonstrated that HNF6 interacts with HBV S promoter and its upregulation leads to suppression of HBV replication and reduced viremia, while HNF6 knockout increased viral loads using HepG2, Huh7 and human embryonic kidney 293T cells [78]. HNF6 induction by upstream proteins has also been confirmed to affect HBV replication. Lahuna et al. suggested that in mice, high levels of CYP2C12 causes upregulation of HNF6, therefore causing suppression of HBV replication [243]. 

#### 3.2.5. Peroxisome Proliferator-Activated Receptor α

Peroxisome Proliferator-Activated Receptor (PPARα) is a liver-enriched nuclear receptor that is associated with beta-oxidation, inflammation, gluconeogenesis, ketone synthesis, fatty acid catabolism, and lipoprotein assembly [244,245,246,247]. PPARα forms a heterodimer with retinoid X receptor (RXRα) and interacts with DNA via its two zinc finger motifs in the DNA-binding domain [248]. The PPARα–RXRα complex activates the ENI, Core and PreS2/S promoter in both hepatic and non-hepatic cell lines [80,249,250]. The activation of these sites via PPARα–RXRα can be further enhanced by the interaction of PGC-1α with this complex [251]. Hu et al. suggested that microRNA-141 interacts with PPARα and inhibits HBV replication in HepG2 cells [252].

#### 3.2.6. Retinoid X Receptor α

Retinoid X Receptor (RXRα) is also a liver-enriched nuclear protein and interacts with other nuclear factors such as COUP-TF, PPARα, and PGC-1α to facilitate binding of genomic DNA and improve transcriptional activation [103,248,251]. It also forms a heterodimer complex with thyroid hormone receptors and retinoic acid receptors [253]. RXRα binds to DNA as a homodimer through RXRα’s DNA-binding domain contains two zinc fingers and three α helices. The C-terminal α helices control the DNA-binding function which recognizes half-sites with the sequence AGGTCA [254]. 

The RXRα-PPARα complex is capable of binding the ENI, core, and S promoter in the HBV genome [81,107]. This complex was shown to enhance HBV replication in HepG2-NTCP cells and other models such as primary Tupaia hepatocytes [255]. The knockdown of RXRα expression decreased infection and cccDNA level as determined by quantitative PCR [255].

#### 3.2.7. Farsenoid X Receptor α

Farsenoid X Receptor α (FXRα) is another liver-enriched nuclear receptor and is a part of the nuclear receptor superfamily of ligand-activated transcription factors [256]. It is activated primarily by bile acids [257] and is associated with cellular processes such as lipid and glucose metabolism as well as liver regeneration [256,258,259,260]. Besides its ligand-binding domain for interacting with bile acids, FXRα has a conserved N-terminal DNA-binding domain that interacts with specific half nuclear receptor sites in the genome (AGGTCA) [261,262]. The FXRα–RXRα complex can bind to the basal core promoter and the ENII site and activate viral transcription as determined using quantitative PCR [66,67]. Moreover, the FXRα–PGC-1α complex has been shown to enhance HBV replication [263]. By using a microRNA-1, FXRα expression can be silenced and allow for the enhancement of HBV replication in HepG2 and Huh7 cell lines [264].

#### 3.2.8. Zinc Finger and Homeoboxes 2 

Zinc finger and homeoboxes 2 (ZHX2) is also a liver-enriched transcriptional repressor in adult hepatocytes and a tumor suppressor [265,266]. It is important for hepatocyte gene regulation and liver development and plays roles in renal cancer and stem cell differentiation [267,268,269,270]. ZHX2 contains four homeodomains and two C2H2 zinc finger motifs that allow for DNA binding causing transcriptional repression as both a homodimer (Figure 3F) or a heterodimer when bound with ZHX1 [265,271]. 

ZHX2 suppresses HBV replication by inhibiting the activity of the X, C and PreS2 promoters [82]. Additionally, ZHX2 also interacts with the A subunit of NF-Y and prevents its DNA binding ability, potentially effecting the recruitment of NF-Y during HBV replication [272]. Interestingly, recent studies demonstrate that HBV infection leads to an over-expression of microRNA-155 which suppresses ZHX2 expression, ultimately accelerating hepatocellular carcinoma progression [273].

#### 3.2.9. Krüppel-Like Factor 15

Krüppel-like factor (KLF15), a liver-enriched transcription factor is associated with cellular functions such as glucose uptake and adipogenesis [274,275,276]. KLF15 contains three C2H2 zinc fingers that facilitate DNA binding to specific GC rich promoter regions (GGGGNGGNG), similar to Sp1 [95,277].

Using ChIP assays, KLF15 has been shown to bind to the S and C promoters and increase HBV transcription [68]. KLF15 increases surface antigen levels by 7-fold in HepG2 cells and 20-fold in Huh7 cell lines. In mice, the use of non-functional KLF15 mutants caused a significant reduction in viral load [68]. Due to the similarities between KLF15 and Sp1, it is hypothesized that the two work synergistically as the S and C promoter regions to facilitate HBV replication [68].

#### 3.2.10. Liver Receptor Homolog 1

Liver receptor homolog 1 (LRH-1) also known as the Fetoprotein transcription factor is associated with liver development and hormonal responses [278,279]. LRH-1 contains both a zinc finger DNA-binding domain and a ligand-binding domain [280]. The DNA-binding domain of LRH-1 recognizes the sequence of YCAAGGYCR while the ligand-binding domains facilitate the binding of other factors such as PROX1 [281,282]. 

It has been demonstrated that LRH-1 binds and activates ENII at two different sites in the HBV genome and activates transcriptional activity. It has also been found to interact with PROX1, HNF1 and HNF4 [69,70]. Specifically, it was suggested that a stronger activation of the ENII site occurs by a synergistic effect of LRH-1 and HNF1 transcription factors [70].

## 4. Conclusions

Many cellular processes modulate the HBV minichromosome (i.e., HBV cccDNA) activity such as cccDNA histone acetylation, epigenetic modifications of the cccDNA and activation of signal transduction pathways such as immune response pathways. For example, IFN-α is an approved treatment for chronic HBV infection, and it was recently found to decrease the number of histones that bind onto the cccDNA, causing repression of HBV replication [55]. Another study by Liu et al. also manipulated the cccDNA by using histone deacetylase (HDAC) inhibitors to disrupt the cccDNA histone acetylation sites. Interestingly, in this study, the HDAC inhibitors increased replication [283]. As the epigenetics has a large regulatory impact on viral replication, specific targeting of epigenetic modifications may be a potential strategy for cccDNA control or elimination. 

Although we have gained an understanding of many aspects of HBV life-cycle, large gaps in knowledge remain in many aspects of HBV replication mechanisms, particularly the HBV cccDNA-host protein interplay. Current oral antivirals (i.e., reverse transcriptase or polymerase inhibitors) suppress HBV replication at downstream stages of replication. These nucleos(t)ide analogs are competitive inhibitors of the HBV polymerase and can downregulate viral replication [90], but the longevity and stability of the cccDNA in the host nucleus allows for recurrence of viremia if therapy is stopped [15]. Furthermore, the viral genome integration into the host genome can contribute to the pool of proteins, putting additional pressure on the immune system, as well as host pathway disruption and carcinogenic potential depending on the site of integrations [8,284]. As the integration of HBV poses another layer of difficulty for effective HBV therapeutics, new approaches may be needed, including other new and emerging technologies in order to completely eradicate HBV. 

Clustered regularly interspaced short palindromic repeats (CRISPR) technology has been recently suggested for therapeutic use. CRISPR/Cas9, an accurate DNA editing technology can be used to mutate the cccDNA in order to destabilize the structure [285]. A recent study by Schiwon et al. demonstrated that the CRISPR/Cas9-based system is capable to inhibit HBV replication [286]. However, the low concentrations of cccDNA in patients limit the effective targeting of CRISPR/Cas9 system, analysis of treatment, and potential off-targeting effects that affect the successful implementation of CRISPR/Cas9 system [285]. 

Gene silencing and/or degradation of mRNA through RNA interference (RNAi) has the potential to diminish cccDNA levels. However, much like CRISPR/Cas9, the accuracy of targeting is a large concern for use as a therapeutic. Furthermore, delivery and the stability of RNAi is also a challenge. One example of how this technology may be implemented is using viral delivery systems that may aid in the deployment of RNAi technologies and ensure accurate targeting to hepatocytes [287,288]. It has been suggested that this technology may be successful as a combinational therapy in order to achieve a more effective outcome [288]. Additionally, the use of N-acetylgalactosamine, a promising technology for liver targeting, in order to deliver RNAi oligonucleotides, can potentially increase the ability for RNAi technology to be taken up into hepatocyte cells without the need of a viral-based delivery mechanism [289].

Finally, the development of therapies that modify the epigenetic profiles of the cccDNA in order to disrupt the structure of the genome can prove to be an effective way of preventing viral load. HBV cccDNA potentially has three methylation regions (CpG islands) which overlap with the ENI, X and core promoter regions [290,291]. Hypermethylation of the CpG regions has been associated with low HBV genomic transcription. Additionally, with methylation events there is a change in acetylation events, leading to the modification of interactions of the cccDNA histone proteins. The general organization of the cccDNA has been described by Tropberger et al. which indicates the potential for epigenetic manipulation [292]. Insights into the epigenetic regulation of the cccDNA and its contributions to HBV replication could aid the development of epigenetic drugs for the treatment of HBV replication. For example, the histone deacetylase SIRT3 has been illustrated to cause a decrease in cccDNA replication. It is hypothesized that SIRT3 manipulates the recruitment of other methyltransferases and therefore changes the overall epigenetic pattern, such that cccDNA is no longer being recognized by host transcription factors [292]. Multiple studies have demonstrated that the histones that package the cccDNA of HBV can have modified activity which can affect overall transcriptional ability [3,9]. Using a specific epigenetic modifier that decreases the stability of the cccDNA, it could be possible to inhibit the overall replication of the virus. Disrupting the stability of cccDNA will also prevent the ability of the transcription factors to bind [293]. Manipulating the structure of the cccDNA might drastically affect the ability of DNA-protein interactions, and therefore for necessary transcription factors required for viral replication. However, this therapy would only prevent viral load and combination therapy may aid with complete eradication of HBV from the patient(s) [290]. 

The current review provides an overview of many transcription factors that can promote and inhibit viral transcription. As pointed out in this review, many transcription factors can promote and inhibit transcription. While such functions could be related to concentration-dependence and/or experimental restraints, it is likely that various protein domains or conformations may also influence these differing functions. These structure-function relationships according to these domains can then be targeted as a potential therapeutic candidate. Imaging methods and binding affinity studies can provide detailed information on the activity as well as the binding characteristics of the interactions. To the best of our knowledge current literature lacks studies of high-resolution structural information on cccDNA–host transcription factor complexes. Using techniques such as small-angle X-ray scattering, cryo-electron microscopy, nuclear magnetic resonance and X-ray crystallography, structural information on individual biomolecules and their complexes can be obtained [53,294,295,296,297]. With such techniques, a better understanding of viral DNA–host protein interactions can be achieved, provide further insights into the HBV replication process, and ultimately guide the development of new therapeutic strategies.

## Figures and Tables

**Table 1:** A list of transcription factors and their effect on viral replication.

Protein	Effect on Transcription	Site of HBV Genome Interaction	References
*Ubiquitous Transcription Factors*			
Activating transcription Factor 2 (ATF2)	Inhibits transcription	Binds ENI region	[33]
Activator Protein 1 (AP-1)	Enhances transcription	Binds PreS2/S region	[34]
CCAAT Enhancer Binding Protein (C/EBP)	Enhances transcription	Binds PreS1 and ENII promoter regions	[5,13,35]
cAMP response element-binding transcription factor (CREB)	Enhances transcription	Binds ENI region	[36,37]
Chicken Ovalbumin Upstream Promoter Transcription Factor (COUP-TF)	Inhibits transcription	Binds ENI, core and PreS2 regions	[13,38,39]
Homeobox A10 (HOXA10)	Inhibits transcription	Binds ENI and X promoter region	[40]
Nuclear Factor kappa B (NF-κB)	Inhibits transcription	Initiate an immune response or is activated by HBx mediated oxidative stress	[41,42]
Nuclear Transcription Factor Y (NF-Y)	Enhances transcription	Binds PreS2/S regions	[4,41,43]
Nuclear Respiratory Factor 1 (NRF1)	Enhanced transcription	Binds X promoter region	[44,45]
Octamer binding protein 1 (Oct1)	Enhances transcription	Binds PreS1 region	[46]
Prospero-related homeobox protein 1 (PROX1)	Inhibits transcription	Binds ENI, ENII and PreS1 regions	[47]
Regulatory Factor Box 1 (RFX1)	Inhibits transcription	Binds ENI and core promoter regions	[48,49,50]
Small heterodimer partner (SHP) ^d^	Inhibits transcription	Binds ENI, ENII, core and X promoter regions	[51]
Specificity protein 1 (Sp1)	Enhances transcription	Binds PreC, ENII and PreS2 promoter regions	[13,23,52,53]
Signal Transducer and Activator of Transcription 1 (STAT1)	Inhibits transcription	Activates an immune response ^a^ binds ENI/X promoter region	[54,55]
Signal Transducer and Activator of Transcription 3 (STAT3)	Enhances transcription	Binds ENI region	[56,57]
TATA Box Protein (TBP)	Enhances transcription	Binds PreS2/S region	[58]
Transcription Factor IIB (TFIIB)	Enhances transcription	Binds X promoter region	[59,60]
Tumor Protein 53 (p53)	Inhibits transcription	Binds ENII and X promoter regions	[61,62,63]
Yin Yang 1 (YY1)	Inhibits transcription ^b^	Binds Upstream of Direct repeat region 1	[64]
Zinc-finger E-box Binding Homeobox 2 (ZEB2)	Inhibits transcription	Binds core promoter region	[65]
*Hepatocyte-specific Transcription Factors*			
Farsenoid X Receptor α (FXRα)	Enhances transcription	Binds ENII and core promoter	[66,67]
Krüppel-like Factor 15 (KLF15)	Enhances transcription	Binds PreS2/S and core promoter	[68]
Liver receptor homolog 1 (LRH-1)	Enhances transcription	Binds ENII region	[69,70]
Hepatocyte Nuclear Factor 1α (HNF1α)	Enhances and inhibits transcription ^c^	Binds PreS1, ENII, core and X promoter regions	[46,71,72]
Hepatocyte Nuclear Factor 3 (HNF3)	Enhances transcription	Binds ENII and PreS1 promoter regions	[38,73,74]
Hepatocyte Nuclear Factor 4 α (HNF4α)	Enhances transcription	Binds PreS1, core and ENII promoter regions	[75,76,77]
Hepatocyte Nuclear Factor 6 (HNF6)	Inhibits transcription	Binds PreS1/S2 promoter regions	[78]
Peroxisome Proliferator-Activated Receptor α (PPARα)	Enhances transcription	Binds ENI, Core and PreS2/S promoter regions	[79,80]
Retinoid X Receptor α (RXRα)	Enhances transcription	Binds ENI, Core and S promoter	[80,81]
Zinc Finger and Homeoboxes 2 (ZHX2)	Inhibits transcription	Binds X, core and PreS2 promoter	[82]

^a^ Through signal transduction STAT1 can inhibit HBV. However, HBx actively prevents its activation, limiting STAT1′s effectiveness against HBV infection. ^b^ With YY1′s interaction with the direct repeat region HBV replication inhibition occurs. However, other studies indicate its important role in integrating the HBV DNA into the host genome. ^c^ HNF1, when interacting with NF-kB, can inhibit HBV replication. With the binding of the ENII, PreS1, core and X promoter, there is an enhancement in activity. ^d^ SHP is recruited by other transcription factors to facilitate HBV replication repression.
